# Trajectory of Change in the Severity of Symptoms in Patients with Fibromyalgia over 24 Months: Exploratory Analyses of a Combination Pharmacological Intervention

**DOI:** 10.3390/jpm14070689

**Published:** 2024-06-26

**Authors:** Fausto Salaffi, Maria Giovanna Lommano, Benedetta Bianchi, Sonia Farah, Francesca Bandinelli, Piercarlo Sarzi-Puttini, Marco Di Carlo

**Affiliations:** 1 Rheumatology Unit, “Carlo Urbani” Hospital, Università Politecnica delle Marche, Jesi, 60035 Ancona, Italy; fausto.salaffi@gmail.com (F.S.); giovanna.lommano@gmail.com (M.G.L.); benedettabianchi.croc@gmail.com (B.B.); sonia.farah91@gmail.com (S.F.); 2 Rheumatology Department, San Giovanni di Dio Hospital, USL Tuscany Center, 50143 Florence, Italy; francesca.bandi@gmail.com; 3 Rheumatology Unit, IRCCS Galeazzi-Sant’Ambrogio Hospital, ASST, School of Medicine, University of Milan, 20157 Milan, Italy; piercarlo.sarziputtini@gmail.com

**Keywords:** fibromyalgia, disease severity, pain magnification, palmitoylethanolamide, acetyl-L-carnitine

## Abstract

Symptoms of fibromyalgia (FM) fluctuate and vary in severity. The current study aimed to evaluate the efficacy of palmitoylethanolamide (PEA) and acetyl-L-carnitine (ALC) in FM patients over a 24-month period and to investigate the mediating function of pain catastrophizing subdomains in unfavorable relationships with disease severity levels in patients with FM. Patients were evaluated at baseline, after 12 months, and after 24 months, using different patient-reported measures (FIQR, FASmod, PSD, and PCS) to distinguish different levels of FM disease severity. A reduction of 30% or more from baseline was considered clinically important (“markedly improved”). A multivariate analysis was performed to identify the variables predictive of an FIQR reduction. Twenty-two patients (28.6%) were classified as “markedly improved”, 16 patients (20.8%) as “slightly/moderately improved”, and 39 patients (50.6%) as “not improved.” The FIQR, FASmod, and PSD scores were significantly reduced at 24 months. The pain magnification domain score of the PCS was the only variable predictive of worse FIQR scores (Wald coefficient: −2.94; *p* = 0.047). These results suggest a potential long-term therapeutic role for the PEA + ALC combination, with pain magnification being the primary predictor of poor efficacy.

## 1. Introduction

Fibromyalgia (FM) is a prevalent chronic pain syndrome distinguished by a well-defined clinical phenotype, which includes widespread pain, tenderness, significant sleep disturbances, fatigue, cognitive dysfunction, and emotional distress [1]. Epidemiological data indicate that FM affects about 6.6% of the global population, with a higher incidence observed in women [2,3]. The symptoms of FM can vary from mild to severe [4], profoundly affecting patients’ personal lives, recreational activities, and occupational performance [5].

Current evidence-based guidelines provide multidisciplinary therapeutic options for FM, available to both patients and healthcare providers [6]. Although pharmacologic interventions such as pregabalin, duloxetine, amitriptyline, and milnacipran are commonly prescribed, their efficacy remains contentious, offering only modest benefits to FM patients [7,8,9]. Longitudinal studies on FM outcomes are sparse. Existing research indicates that complete remission is rare, although some patients may notice fluctuating symptom patterns or temporary improvements [10,11,12]. For instance, a longitudinal study tracking established FM patients (median disease duration at initial assessment: 7.8 years) reported increased functional disability over 7 years, although other metrics like pain severity, global symptom severity, fatigue, sleep quality, anxiety, depression, and overall health status remained stable. Remarkably, patient satisfaction with health outcomes showed improvement [10]. In contrast, studies in the US and the UK reflect mixed results regarding symptom progression over the years following diagnosis [12,13,14].

There is also evidence suggesting that 20–44% of individuals previously diagnosed with FM may not meet the clinical criteria for the condition in subsequent evaluations [13,15]. Due to the limited efficacy and potential adverse effects of pregabalin and duloxetine at therapeutic doses, most patients derive only partial relief, often necessitating combination therapy in clinical settings. Despite this, earlier guidelines from leading pain and rheumatology societies did not endorse or oppose such combination pharmacotherapy for FM [16,17,18]. More recent recommendations from the Canadian Pain Society and the Canadian Rheumatology Association, however, advocate for a pharmacological regimen that simultaneously addresses multiple symptoms and may involve a combination of drugs, with careful consideration of drug interactions (level 5, grade D) [19]. The Italian Society for Rheumatology similarly supports a multimodal therapeutic strategy that includes drug combinations [20].

In this context, the integration of pharmaceuticals and nutraceuticals, such as palmitoylethanolamide (PEA) and acetyl-L-carnitine (ALC), has shown promise [21]. PEA has been investigated for its analgesic and anti-inflammatory properties in FM, functioning as an endogenous modulator of inflammation and nociception [22]. ALC, uniquely, exhibits analgesic effects mediated through an epigenetic mechanism involving the acetylation of p65/RelA, a key transcription factor in the NFkB pathway [23].

Given the scarcity of longitudinal data on FM patient outcomes, this study aims to: (i) delineate symptom trajectory groups among FM patients supplemented with PEA and ALC over a 2-year follow-up and (ii) identify the variables predictive of worse outcomes.

## 2. Materials and Methods

### 2.1. Patients

This study involved a retrospective extraction of data from a comprehensive database of patients registered in the Italian Fibromyalgia Registry (IFR) [14]. The IFR serves solely as a basis for the collection of clinical and clinimetric data. The inclusion of patients in the IFR does not, in itself, involve any predefined therapeutic intervention. The patients with FM included in this study were extracted from those enrolled in a multidisciplinary treatment program, treated between November 2018 and February 2024. The patients are affiliated with the Rheumatology Unit of “Carlo Urbani” Hospital in Jesi, Università Politecnica delle Marche. To date, the IFR includes over 1300 patients from “Carlo Urbani” Hospital.

Eligibility for participation was determined according to the American College of Rheumatology (ACR) 2016 criteria for FM [24,25]. In addition to meeting the ACR 2016 criteria, the retrospective inclusion involved patients treated with a combination of PEA (600 mg BID) and ALC (500 mg BID). This treatment regimen was added to usual care for a period of 24 months. The included patients were evaluated at three predetermined time points: baseline (the start of the PEA + ALC combination treatment), after 12 months, and after 24 months.

The ACR 2016 criteria for FM involve a two-part assessment. Initially, patients identify pain sites across 19 specific body areas distributed over five regions: the upper left region includes the left jaw, shoulder girdle, upper arm, and lower arm; the upper right region includes the right jaw, shoulder girdle, upper arm, and lower arm; the lower left region covers the left hip, upper leg, and lower leg; and the lower right region includes the right hip, upper leg, and lower leg. The Widespread Pain Index (WPI) is calculated based on the number of these regions experiencing pain. For an FM diagnosis, the patient must report pain in at least four out of the five regions as specified by the criteria. The second component evaluates the intensity of several symptoms including fatigue, waking unrefreshed, cognitive disturbances, as well as headache, abdominal cramps, and depression over the previous 6 months, each rated on a scale from 0 to 3. This forms the Symptom Severity Scale (SSS). A diagnosis of FM is confirmed if the WPI is ≥7 and the SSS is ≥5, or if the WPI ranges from 4 to 6 and the SSS is ≥9 [24,25]. Only patients who completed both baseline and follow-up assessments were included in the analysis. Details of the study methods and baseline findings have been published previously [26]. Diagnosis of FM was conducted by a rheumatologist with at least 10 years of experience. All participants underwent a thorough diagnostic evaluation, which included a comprehensive physical examination and laboratory tests in line with the latest recommendations by the European Alliance of Associations for Rheumatology (EULAR) for FM management [27].

Exclusion criteria included major concurrent psychological disorders such as severe depression, coexisting connective tissue diseases, inflammatory arthropathies, uncontrolled hypertension, diabetes, HIV, narrow-angle glaucoma, or malignancies that could confound the FM assessment metrics. Additionally, individuals with a history of significant abuse of illicit drugs, prescription medications, or alcohol, or those consuming more than 200 mg of oral morphine equivalents per day were also excluded.

### 2.2. Measurements and Instruments

The clinimetric assessment was identical for all three visits (baseline, after 12 months, and after 24 months) and was based on tools that currently serve as international standards for evaluating the severity of FM. A series of questionnaires incorporating several validated patient-reported outcome (PRO) instruments were administered to patients: the revised Fibromyalgia Impact Questionnaire (FIQR) [28], the modified Fibromyalgia Assessment Status (FASmod) [29,30], the PolySymptomatic Distress Scale (PSD) [31], and the Pain Catastrophizing Scale (PCS) [32,33]. Essential sociodemographic variables collected included age, sex, body mass index (BMI), and level of formal education (primary school, middle school, high school/university).

The FIQR, an updated version of the Fibromyalgia Impact Questionnaire (FIQ) designed to address limitations of the original instrument, comprises twenty-one 0–10 numerical rating scales (NRS, with 10 indicating the “worst”). It evaluates three primary health domains: function, overall impact, and symptoms, with all questions referring to the previous 7 days. The final score ranges from 0 to 100, where higher values indicate greater disease severity. This score is computed by dividing the 9-item function domain total (range 0–90) by three, directly using the 2-item overall impact domain total (range 0–20), and halving the 10-item symptom domain total (range 0–100). These three sub-scores are then summed. Proposed cut-off points for disease severity are: 0–23 for remission, 24–40 for mild disease, 41–63 for moderate disease, 64–82 for severe disease, and 83–100 for very severe disease [4].

The FASmod consists of two sections [30]. The first includes two questions regarding fatigue and unrefreshing sleep over the prior week, with each item rated on a 0–10 NRS. The maximum sub-score for the first section is 20. The second section employs a front-back mannequin indicating 19 body areas where patients mark their pain, scoring 1 point per area. The total FASmod score ranges from 0 to 39, with severity cut-offs at 0–12 for remission, 13–20 for mild disease, 21–28 for moderate disease, 29–33 for severe disease, and 34–39 for very severe disease [4].

Derived from variables used in the 2010/2011 ACR criteria as modified for surveys and clinical research, the PSD includes the WPI and the SSS for determination [33]. PSD severity cut-off points are 0–5 for remission, 6–15 for mild disease, 16–20 for moderate disease, 21–25 for severe disease, and 26–31 for very severe disease [4].

The PCS, a 13-item self-report questionnaire commonly used in chronic pain research and clinical settings, probes respondents’ typical thoughts and feelings when confronted with pain cues. It utilizes a 0–4 Likert scale for respondents to rate the frequency of each item (0 = never; 4 = always). The total PCS score and the sub-scores for pain magnification (items 6, 7, and 13), rumination (items 8 through 11), and helplessness (items 1 through 5 and 12) are calculated. A total PCS score of 30 or more is considered indicative of catastrophizing [32]. The PCS has been successfully translated into Italian (PCS-I) [33]. PCS-I exhibited psychometric properties consistent with earlier versions [32].

### 2.3. Statistical Analysis

Descriptive statistics were computed for all variables in the study. These included mean values, standard deviations (SDs), medians, and interquartile ranges (IQRs) for continuous variables, as well as frequencies and percentages for categorical variables. The normality of distribution for each variable was assessed using the Shapiro–Wilk test.

Changes in the FIQR, the FASmod, and the PSD total scores were quantified using the formula: [(follow-up score − baseline score)/baseline score] × 100. A change of 30% or more from the baseline was deemed clinically significant [23]. Patients with an improvement of 30% or greater in all three clinimetric indices were classified as “markedly improved”, those with an improvement between 1% and 29% as “slightly/moderately improved”, and those with no improvement as “not improved.”

The Kruskal–Wallis test was utilized to analyze the significance of changes in scores from baseline to the 24-month follow-up period.

Finally, a multivariate analysis was conducted to identify predictors of improved disease outcomes, with the reduction in FIQR scores as the dependent variable. Independent variables included changes in pain-related indices such as PCS magnification, PCS helplessness, PCS rumination, and WPI), changes in symptoms domain (FAS fatigue, FAS unrefreshing sleep, and SSS), and demographic characteristics (age, BMI, duration of disease, education level).

Data were managed using a Microsoft Excel database, and statistical evaluation was performed using MedCalc^®^ software, version 20.07 (MedCalc Software, Mariakerke, Belgium).

## 3. Results

### 3.1. Study Sample Characteristics

The study cohort consisted of 77 patients diagnosed with FM, comprising 68 women (93.7%) and 9 men (6.3%). The mean age of participants was 53.4 years (SD 12.2 years), and the mean duration of disease was 7.5 years (SD 5.2 years). A significant majority, 75.3%, of the patients were married, and most had attained an education level of high school or higher. The group had a mean BMI of 28.5 kg/m^2^ (SD 4.8 kg/m^2^), classifying them as moderately overweight.

Table 1 presents key demographic and clinimetric data, including scores from the FIQR and the PCS, for the entire cohort.

The baseline patient-reported outcomes underscore a persistently high disease burden among participants. Notably, the median baseline FIQR was 61.5 (IQR 51.0–75.8), the FASmod was 32.6 (IQR 28.0–35.0), and the PSD was 27.0 (IQR 21.5–40.0).

### 3.2. Patterns of Treatment

In addition to the previously mentioned PEA + ALC combination, the utilization of prescription medications for FM remained relatively consistent across the three assessments, with 60.2%, 62.0%, and 63.1% of patients using them during the follow-up period. The predominant class of medications reported in the FM cohort at follow-up included serotonin and norepinephrine reuptake inhibitors (SNRIs), selective serotonin reuptake inhibitors (SSRIs), tramadol, antiepileptics (notably pregabalin), nonsteroidal anti-inflammatory drugs (NSAIDs), and acetaminophen, each exceeding a 10% usage rate. Although there were fluctuations in the usage of different medication classes for pain management, none of these changes reached statistical significance. Notably, NSAIDs and muscle relaxants (specifically cyclobenzaprine) experienced the most significant shifts, with at least a 5-percentage point increase in their rates of use.

In terms of nonprescription pain relief, a substantial majority of patients reported using over-the-counter medications, with 84.2% at baseline, decreasing slightly to 80.9% at the 12-month follow-up and 81.7% at the 24-month follow-up. The use of supplements such as other pain relief supplements including cannabis sativa (comprising both cannabidiol and tetrahydrocannabinol) declined from 34.9% at baseline to 27.5% by the 24-month follow-up.

Conversely, there was a notable increase in the percentage of patients engaging in physical treatments, rising from 28.9% at baseline to 39.4% at the 24-month follow-up. This shift indicates a growing inclination towards non-pharmacological interventions for managing FM symptoms over time.

### 3.3. Trajectories of Symptom Severity

In this exploratory analysis, we identified three distinct trajectories of symptom severity among FM patients, based on the changes observed in their clinical scores over time. A total of 22 patients (28.6%) were classified as “markedly improved”, 16 patients (20.8%) as “slightly/moderately improved”, and 39 patients (50.6%) as “not improved.”

The changes in the FIQR total scores for these groups were as follows: for the “markedly improved” group, there was a decrease of 51.9% (95% CI: −59.1% to −44.8%); for the “slightly/moderately improved” group, a decrease of 18.3% (95% CI: −21.2% to −15.3%); and for the “not improved” group, an increase of 11.1% (95% CI: 2.9% to 19.3%). The statistical analysis revealed significant differences between these groups (Ht = 63.7; *p* < 0.0001) (Table 2).

Regarding the FASmod, the respective changes were −46.6% (95% CI: −54.0% to −39.3%), −23.5% (95% CI: −33.1% to −13.9%), and −12.0% (95% CI: −18.2% to −5.8%), with the analysis showing significant differences (Ht = 34.9; *p* < 0.0001) among groups (Table 3). The PSD followed a similar pattern, with reductions of 41.8% (95% CI: −53.9% to −29.8%) and 13.2% (95% CI: −36.8% to −10.4%) in the first two groups, and an increase of 11.1% (95% CI: −7.5% to 29.7%) in the “not improved” group (Ht = 23.3; *p* < 0.0001) (Table 4).

**Figure 1:**
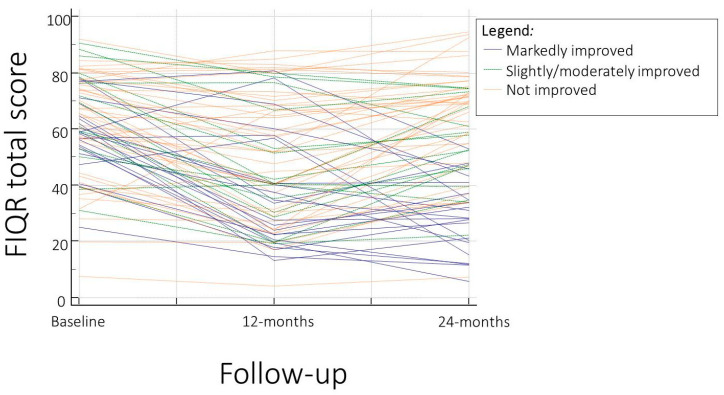
Trajectories of the three distinct symptom severity patterns according to the revised Fibromyalgia Impact Questionnaire (FIQR) total score.

Figure 1 in this report illustrates the trajectories of the FIQR, FASmod, and PSD total scores, measured at three time points across the entire cohort of FM patients. This visual representation highlights the differential progression of symptoms severity across the study population.

### 3.4. Predictors and Associated Factors of Long-Term FIQR Total Score

In examining the long-term predictors of the FIQR total score, our analysis focused on identifying variables that could be linked to the persistence of disease severity. During the process of variable selection, the pain magnification domain score of the PCS emerged as the most significant factor associated with ongoing disease severity (Wald coefficient = 2.94, *p* = 0.047) (Table 5).

Conversely, our study found no significant predisposing effects over a 2-year period for several other potential predictors. Specifically, the WPI, SSS, FASmod scores related to unrefreshing sleep and fatigue, as well as BMI showed no significant association with the long-term FIQR total score. Additionally, demographic factors such as age, symptom duration, and level of education were not predictive of the long-term FIQR total score.

## 4. Discussion

This study, which followed FM patients for a significant period (24 months) and described symptom severity trajectories, demonstrated that combination therapy with PEA and ALC results in significant improvement in 28.6% of patients. The only clinical predictor associated with non-response is baseline pain magnification.

Management of FM poses significant challenges, given the complexity of its symptoms and the variable efficacy of treatments [34]. Multidisciplinary approaches combining pharmacological and non-pharmacological therapies have been validated in clinical trials spanning 3 to 12 months, showing effective outcomes [35,36]. International guidelines recommend multi-component intervention programs, which endorse therapies including pharmacological treatments, mindfulness, hydrotherapy, and acupuncture [7,37,38]. These approaches emphasize the potential benefits of complementary therapies in managing FM.

Despite the predominance of pharmacotherapy in managing FM, the efficacy of such treatments is often partial and accompanied by side effects at therapeutic dosages, leading to incomplete relief in many patients [39]. Indeed, about 50% of patients do not show significant improvement with pharmacological treatments alone [40], leading to frequent use of combination therapy involving drugs like pregabalin (PGB) and duloxetine (DLX) in clinical settings.

Recent guidelines from the Canadian Pain Society and Canadian Rheumatology Association highlight the necessity of selecting pharmacological agents that can concurrently manage multiple symptoms, possibly requiring a combination of medications. Such combinations necessitate careful consideration of drug interactions (Level 5, Grade D) [19]. This stance is echoed by the expert panel of the Italian Society for Rheumatology, which supports a multimodal approach that includes drug combinations [20].

The integration of nutraceuticals like PEA and ALC with drugs also offers promising outcomes. PEA is recognized for its analgesic and anti-inflammatory effects, acting through multiple pathways including the CB2-like receptor, GPR-55, and PPAR family receptors, along with inhibiting mast cell degranulation—a process termed Autacoid Local Inflammation Antagonism (ALIA) [41]. PEA’s effects were specifically explored in FM in a dedicated study [22].

ALC offers another approach to nociplastic pain therapy by enhancing the effects of nerve growth factor (NGF) and contributing to mitochondrial energy homeostasis and detoxification [42]. Its antinociceptive properties, demonstrated in several models of neuropathic pain, involve epigenetic mechanisms related to the acetylation of p65/RelA, a key transcription factor of the NFkB family. ALC also influences the dorsal root ganglia and dorsal horns of the spinal cord by increasing the expression of metabotropic glutamate receptor type 2 (mGlu2), thereby reducing glutamate release from sensory fibers. Furthermore, due to its structural similarity to acetylcholine, ALC may enhance acetyl-CoA absorption into mitochondria and exhibit cholinomimetic effects [23].

In this exploratory study, we analyzed repeated self-reported FIQR, FASmod, and PSD total scores to delineate potential symptom trajectories in FM patients. We identified three distinct trajectory groups, termed “no improvement”, “some improvement”, and “marked improvement.” Notably, 49.4% of the participants reported a significant change in overall symptom severity over time, with a trend towards improvement. No significant differences in baseline characteristics were observed between the trajectory groups. These findings align with previous research, suggesting that FM symptoms can fluctuate, and some patients may experience symptom improvements following diagnosis and treatment.

A prior study reported that 66% of FM patients in rheumatology clinics experienced slight to significant improvement in their symptoms 10 years post-diagnosis [12]. Such improvements were associated with younger age and shorter symptom duration at diagnosis. Conversely, another study found that 47% of FM outpatients reported moderate to marked improvements over 3 years [19]. Literature suggests better outcomes for FM individuals in community settings than those seen in rheumatology clinics [43]. However, these results contrast with an observational study that followed 1555 FM patients using semi-annual questionnaires for up to 11 years [44], where most participants reported persistent high symptom levels and only 25% noted a slight improvement trend. Another longitudinal study evaluating FM patients at two time points over 2 years observed consistently high levels of disease burden [45].

Our study participants reported fluctuating FM symptoms and transitions between different symptom severity categories over time, a finding echoed in other longitudinal studies [46]. To our knowledge, our study is the first to provide insights into symptom intensity and changes over a 24-month period, enhancing our understanding of symptom variability in FM.

FM is a complex condition requiring a comprehensive assessment strategy to understand and manage its myriad symptoms. Unlike previous studies that focused on clusters of 2–3 symptoms [47,48], our study considered a wider range of symptoms, providing a more representative view of the overall symptom burden in FM. For instance, we noted that changes in catastrophizing could predict alterations in anxiety and depression, which may subsequently influence a decline in patient-reported pain, unrefreshing sleep, and fatigue.

Catastrophizing is defined as “an excessive negative mental attitude brought to bear during a real or expected painful experience” [32]. This cognitive process, known as pain catastrophizing, is recognized as one of the most potent psychological factors contributing to poor pain outcomes. It has been characterized as a behavioral pattern of repetitive negative thought, serving as a stabilizing mechanism that helps individuals manage painful internal experiences. Catastrophizing is notably predictive of disability, pain, and sickness behavior over time, often manifesting through negative cognitive distortions about the significance of pain and its potential consequences [49].

The PCS, developed by Sullivan et al., comprises items specifically tailored to assess various dimensions of pain catastrophizing [32]. Initial factor analysis identified three domains of catastrophizing within the PCS, and subsequent exploratory and confirmatory factor-analytic studies in patient populations have largely corroborated this three-factor structure. Of these, the helplessness and rumination domains have consistently shown stronger associations with pain severity and pain-related functional impairments than scores on the magnification scale [50]. Notably, the helplessness domain of the PCS is associated with poorer psychological outcomes [10], greater pain intensity [11], and increased pain interference [12], compared to the other dimensions.

Consequently, an inability to manage pain-related catastrophizing emerges as a critical factor strongly linked to the prevalence of chronic pain. This aligns with our findings that pain-related catastrophizing significantly correlates with the occurrence of chronic pain in community-dwelling elderly individuals. Furthermore, in a cross-sectional study, patients with FM possessing the BDNF Val66Met (rs6265) polymorphism exhibited heightened levels of pain catastrophizing. This genotype was significantly associated with increased scores on the magnification and rumination dimensions of the bodily pain PCS (BP-PCS), with statistical significance (*p* < 0.05) [51]. Additionally, Rodero et al. demonstrated that the magnification and helplessness dimensions predict the impact of FM beyond the variance explained by pain severity in patients diagnosed for 2–4 years [52].

The importance of pain catastrophizing, in terms of pain magnification, has also emerged in the context of complementary treatments of FM. A study published in 2023 identified pain magnification, along with myofascial tender point count, as the only two clinical variables predictive of nonresponse to a course of acupuncture treatment in patients with FM [53].

The regression analysis indicated that exploring the relationships between the subdomains of catastrophizing and the total scores on the FIQR could provide deeper insights. Notably, the magnification domain of catastrophizing was found to be most strongly associated with the FIQR scores. However, it remains uncertain whether these findings regarding catastrophizing subdomains would replicate in a diverse patient sample spanning various cultures or languages.

Current treatments for FM are generally ineffective, and the variability of symptoms among individuals complicates this issue further. When patients present at clinics, physicians often employ their own ad hoc methods for assessing symptoms and selecting treatments. Recognizing the variability of symptoms could enable clinicians to better understand and track the relationships between different symptoms over time. This understanding could pave the way for more effective and comprehensive treatment strategies. Given the observed variability in symptom intensity and its fluctuations during the 3-month study period, it is crucial for researchers to accurately model the symptom experience in FM. Similarly, clinicians should consider the implications of these and other findings in formulating treatment approaches.

This study did have several limitations that warrant mention. The small sample size limits the statistical power and reduces the generalizability of the results to a wider FM patient population. Additionally, the absence of a control group of non-FM patients is another limitation. The study employed a basic cross-sectional design to identify characteristics influencing symptom manifestation. Although the primary goal of this study was to assess feasibility and provide descriptive data for potential future studies employing intensive longitudinal designs, the lack of comparative groups, such as other patient cohorts or healthy controls, is a limitation. Future research should explore symptom experience differences not only between FM patients and healthy controls but also among patients with other chronic conditions such as arthritis, diabetes, and cancer. In these conditions, ongoing pain and fatigue are common symptoms that pose a significant and often unpredictable challenge to daily life quality.

## 5. Conclusions

More than a quarter of patients undergoing combination therapy with PEA and ALC for 24 months, in addition to usual care, achieve significant improvement in the severity of FM symptoms. However, approximately half of the patients do not show any improvement. Among the clinical variables studied, high levels of pain magnification are the primary predictors of non-response to the treatment. These findings may be useful in guiding therapeutic choices and emphasize the necessity of assessing clinical aspects of the disease, such as pain catastrophizing, which can be crucial in determining the response to a specific treatment. This approach can help avoid additional burdens for patients caused by ineffective and costly treatments.

## Figures and Tables

**Table 1:** Mean, standard deviations (SDs), median, and interquartile (IQR) range at baseline and during follow-up of the clinimetric assessment.

	Assessments											
	Baseline				12 Months				24 Months			
	Mean	Median	SD	IQR	Mean	Median	SD	IQR	Mean	Median	SD	IQR
FIQR physical domain	17.50	17.30	6.02	14.00–22.77	14.85	14.70	11.37	6.30–21.07	16.70	15.60	17.44	9.25–20.70
FIQR general status domain	11.42	12.00	5.04	7.00–15.25	7.75	7.00	5.57	3.00–12.25	9.24	10.00	6.15	4.00–14.00
FIQR symptoms domain	31.82	32.50	8.97	26.75–38.50	24.65	24.00	10.36	16.50–32.62	27.92	29.50	10.75	19.87–37.00
FIQR total score	60.74	61.50	17.88	51.00–75.82	47.25	45.60	22.56	25.47–66.87	53.86	53.00	22.77	34.00–71.65
FASmod unrefreshing sleep	6.75	8.00	2.94	4.00–9.00	5.29	5.00	2.86	3.00–8.00	6.11	7.00	2.80	4.00–8.00
FASmod fatigue	7.76	8.00	1.97	7.00–9.00	6.14	7.00	2.48	4.00–8.00	6.65	7.00	2.42	4.00–9.00
FASmod WPI	18.06	17.00	1.09	16.00–18.00	13.75	12.00	5.31	8.00–14.25	12.06	11.50	4.90	8.00–14.00
FASmod total score	32.57	33.00	4.67	28.00–35.00	25.18	24.00	8.08	14.75–27.25	24.82	24.00	7.85	18.75–30.00
SSS score	8.95	8.00	5.22	4.00–10.00	8.91	8.00	4.98	5.00–10.00	10.50	10.00	4.76	5.50–10.50
PSD total score	27.01	29.00	9.95	18.00–37.00	22.66	23.00	9.98	15.00–31.25	22.56	25.00	10.87	14.75–32.00
PCS helplessness subscale	12.96	13.00	5.66	8.00–17.00	9.45	9.00	5.30	4.75–14.00	10.05	10.00	5.68	5.00–14.25
PCS magnification subscale	3.96	5.00	2.12	2.00–6.00	3.45	3.00	2.25	1.00–5.00	3.48	3.00	2.38	1.00–6.00
PCS rumination subscale	12.57	13.00	4.79	9.00–16.50	10.37	10.00	4.36	8.00–14.00	10.37	11.00	5.14	6.75–14.00
PCS total score	29.49	30.00	11.73	21.50–40.00	23.27	22.00	10.81	15.00–32.50	23.90	25.00	12.26	14.00–34.00

Abbreviations: SD = standard deviation; FIQR = revised Fibromyalgia Impact Questionnaire; FASmod = modified Fibromyalgia Assessment Status; WPI = Widespread Pain Index; SSS = Symptom Severity Scale; PSD = PolySymptomatic Distress Scale; PCS = Pain Catastrophizing Scale.

**Table 2:** Levels of change of the FIQR total score for the three groups and differences (Kruskal Wallis test).

Variable Y (Data)	FIQR Total Score					
Summary Measure of Interest	% Difference Last-First					
Group	n	Mean	95% CI	SD	Median	95% CI
Markedly improved	22	−51.93	−59.13 – −44.79	16.24	−48.85	−63.87 – −41.12
Slightly/moderately improved	16	−18.27	−21.26 – −15.29	5.59	−17.90	−21.76 – −13.12
Not improved	39	11.10	2.93 – 19.28	25.21	2.89	−1.93 – 7.04
Kruskal-Wallis test						
Factor	n	Average Rank				
Markedly improved	22	11.50				
Slightly/moderately improved	16	30.50				
Not improved	39	58.00				
Test statistic	63.68					
Corrected for ties Ht	63.68					
Degrees of Freedom (DF)	2					
Significance level	*p* < 0.0001					

Abbreviations: FIQR = revised Fibromyalgia Impact Questionnaire; CI = confidence interval; SD = standard deviation.

**Table 3:** Levels of change of the FASmod total score for the three groups and differences (Kruskal Wallis test).

Variable Y (Data)	FASmod Total Score					
Summary Measure of Interest	% Difference Last-First					
Group	n	Mean	95% CI	SD	Median	95% CI
Markedly improved	22	−46.66	−54.01 – −39.31	16.56	−41.74	−54.28–−35.89
Slightly/moderately improved	16	−23.52	−33.11 – −13.93	17.99	−25.29	−37.87 – −8.06
Not improved	39	−12.03	−18.24 – −5.83	19.13	−11.53	−17.94 – −2.85
Kruskal-Wallis test						
Factor	n	Average Rank				
Markedly improved	22	16.59				
Slightly/moderately improved	16	38.59				
Not improved	39	51.81				
Test statistic	34.86					
Corrected for ties Ht	34.87					
Degrees of Freedom (DF)	2					
Significance level	*p* < 0.0001					

Abbreviations: FASmod = modified Fibromyalgia Assessment Status; CI = confidence interval; SD = standard deviation.

**Table 4:** Levels of change of the PSD total score for the three groups and differences (Kruskal Wallis test).

Variable Y (Data)	PSD Total Score					
Summary Measure of Interest	% Difference Last-First					
Group	n	Mean	95% CI	SD	Median	95% CI
Markedly improved	22	−41.85	−53.90 – −29.79	27.18	−43.00	−55.94 – −27.74
Slightly/moderately improved	16	−13.20	−36.79 – 10.38	44.27	−16.75	−39.67 – −3.27
Not improved	39	11.12	−7.48 – 29.73	57.40	−2.63	−6.26 – 5.70
Kruskal-Wallis test						
Factor	n	Average Rank				
Markedly improved	22	21.23				
Slightly/moderately improved	16	36.88				
Not improved	39	49.90				
Test statistic	23.28					
Corrected for ties Ht	23.28					
Degrees of Freedom (DF)	2					
Significance level	*p* < 0.0001					

Abbreviations: PSD = PolySymptomatic Distress Scale; CI = confidence interval; SD = standard deviation.

**Table 5:** Multivariate analysis of the variables predictive of the FIQR total score. Contingency table for Hosmer and Lemeshow test [Show].

Variable	Coefficient	Standard Error	Wald	*p*
Age (years)	−0.008	0.010	0.627	0.428
BMI (kg/m^2^)	−0.019	0.010	3.397	0.065
Disease duration (years)	−0.051	0.048	1.198	0.277
Level of education (years)	−0.000	0.038	0.000	0.989
FIQR physical domain	−0.044	0.039	1.237	0.265
FASmod unrefreshing sleep	−0.006	0.058	0.017	0.913
FASmod fatigue	0.037	0.032	1.317	0.251
WPI score	−0.102	0.083	1.722	0.189
SSS score	−0.024	0.024	1.466	0.225
PCS helplessness subscale	0.001	0.052	0.001	0.971
PCS magnification subscale *	−0.224	0.079	2.942	0.047
PCS rumination subscale	0.004	0.053	0.774	0.378
Constant	1.589	0.565	7.898	0.004
Null model −2 Log Likelihood	206.551			
Full model −2 Log Likelihood	177.839			
Chi-squared	28.713			
Significance level	*p* = 0.0004			

Abbreviations and legend: FIQR = revised Fibromyalgia Impact Questionnaire; FASmod = modified Fibromyalgia Assessment Status; WPI = Widespread Pain Index; SSS = Symptom Severity Scale; PCS = Pain Catastrophizing Scale; * = significative variable.

## Data Availability

Study data are available upon reasonable request to the corresponding author.
